# Content validity and test-retest reliability of patient perception of intensity of urgency scale (PPIUS) for overactive bladder

**DOI:** 10.1186/1471-2490-12-26

**Published:** 2012-09-07

**Authors:** Sherilyn M Notte, Thomas S Marshall, Misun Lee, Zalmai Hakimi, Isaac Odeyemi, Wen-Hung Chen, Dennis A Revicki

**Affiliations:** 1 United BioSource Corporation, 7101 Wisconsin Ave, Suite 600, Bethesda, MD, 20814, USA; 2 Astellas Pharma US, 3 Parkway North, Deerfield, IL, 60015, USA; 3 Astellas Pharma Global Development, 3 Parkway North, Deerfield, IL, 60015, USA; 4 Astellas Pharma Global Development, Elisabethhof 19, 2353, EW Leiderdorp, The Netherlands; 5 Astellas Pharma Europe Ltd, Lovett House, Lovett Road, Staines, Middlesex, TW18 3AZ, UK

**Keywords:** Over active bladder, OAB, Urinary urgency, Urge incontinence, Patient perception of intensity of urgency scale, PPIUS

## Abstract

**Background:**

The Patient Perception of Intensity of Urgency Scale (PPIUS) is a patient-reported outcome instrument intended to measure the intensity of urgency associated with each urinary or incontinence episode. The objectives of this study were to assess the content validity, test-retest reliability, and acclimation effect of the PPIUS in overactive bladder (OAB) patients.

**Methods:**

Patients undergoing treatment for OAB were recruited to participate in a non-interventional study by completing a three-day micturition diary including the PPIUS for three consecutive weeks. Following completion of the three-week study, participants from two select sites also completed a cognitive interview to assess their comprehension of the PPIUS.

**Results:**

Thirty-nine participants successfully completed the three-week test-retest study; twelve of these participants completed the cognitive interview. Test-retest reliability was high based on intra-class correlation coefficient of 0.95. Among stable patients, the difference between the mean ratings of any two weeks was non-significant. Among the twelve interview participants, nine found it simple to choose a PPIUS rating for each of their micturition episodes and most found the urgency rating definitions consistent with their urgency experiences.

**Conclusions:**

The results demonstrated content validity based on qualitative interviews, and excellent test-retest reliability among stable patients. In addition, no acclimation effect was observed among stable patients. These findings support the use of the PPIUS as a reliable measure of urgency in both clinical trial and real life settings. The validity of PPIUS could be further established with future studies investigating the relationship between discretely graded urgency and incontinence continuum.

## Background

Overactive bladder syndrome (OAB), according to the Standardization Subcommittee of the International Continence Society (ICS), is a symptom-defined condition characterized by urinary urgency, usually with urinary frequency and nocturia, and may or may not be accompanied by urge incontinence [1]. OAB has been shown to cause significant symptomatic burden and disrupt daily activities, sleep, and personal relationships [2]. It is common in clinical practice to ask patients to use a diary to record urinary episodes of their OAB symptoms. However, few existing questionnaires ask about the intensity of the urgency, one of the key symptoms of OAB, which can have as much of an impact as the frequency of urinary urgency [2].

The Patient Perception of Intensity and Urgency Scale (PPIUS) has been recommended when assessing the degree of urgency felt by patients at each micturition [3]. The PPIUS is a 5-point scale designed for measurement of both urinary urgency and urge incontinence. It has been incorporated into a micturition diary and has been used in OAB clinical trials [4-6]. Cartwright, Srikrishna, Cardazo, & Robinson [7] found that the PPIUS has good reliability, excellent known groups validity and convergent validity, and high responsiveness when used in a clinical trial study with adult women with OAB. The test-retest reliability of the PPIUS incorporated into a seven-week diary completed before and after a one-week interval has been reported in a group of asymptomatic women [8]. However, content validity has yet to be supported with qualitative research in patients with OAB. Furthermore, the possibility of an acclimation effect has not been examined among patients with OAB. Acclimation effects exist when data initially collected (i.e., the first week) are significantly different from data collected after patients become familiar with the diary, rendering the initial data unreliable.

The objective of this study was to assess the content validity of the PPIUS, the test-retest reliability of the mean PPIUS urgency ratings in men and women with symptoms of OAB, and whether OAB patients demonstrate an acclimation effect when completing the PPIUS for three weeks.

## Methods

### Study design

Males and females over the age of 18 years old with clinical records that described symptoms of OAB for at least three months and on continuous treatment for OAB for at least three months (based on clinician-report/prescription) were eligible for this study. These symptoms included frequency, urgency, nocturia, and/or urge incontinence. The patients were not required to undergo the urodynamic testing (UDS) for this study. Exclusion criteria were as follows: pregnant or breastfeeding women; significant stress incontinence or mixed stress/urge incontinence where stress is the predominant factor as determined by site study staff; self-catheterization; diabetic neuropathy; urinary tract infection (confirmed by dipstick test); non-drug treatment, including electro-stimulation therapy; any clinically significant condition or visual or cognitive impairment which in the opinion of the investigator makes the subject unsuitable for the study. Patients were recruited from five clinical sites in the different states in the United States (US): Oklahoma, California, Utah, Ohio, and Massachusetts. Recruitment was on a continuous basis and lasted approximately two months until recruitment goals were met. The study protocol was approved by Ethical Review Committee, Inc. (Independence, MO) (ERC ID #485-07-09). In addition, all recruitment procedures met Health Insurance Portability and Accountability Act of 1996 requirements and approval standards, and all applicable state and federal laws and regulations. An estimated sample size of 36 stable participants was required in order to achieve 90% statistical power at a 2-sided 5% significance test for an intra-class correlation coefficient (ICC) of 0.80 versus 0.50 [9].

Patients were first recruited to participate in a two-visit non-interventional study to assess the test-retest reliability of the PPIUS. Following completion of the two-visit study, successful completers of the test-retest study from two geographically diverse and high-recruiting sites were asked to participate in a third visit, consisting of a one-on-one cognitive interview to evaluate their comprehension and understanding of the PPIUS, and provide evidence of content validity. Participants from the two selected sites who agreed to participate in the cognitive interview were scheduled 1–2 weeks after they completed the test-retest study.

At the first clinic visit, participants were trained by site study staff on how to complete the PPIUS as a part of a three-day diary at home over the following three consecutive weeks. Participants mailed back the Week 1 and Week 2 diaries to the clinical site immediately upon completion, and returned the Week 3 PPIUS diary to the site at the second clinic visit (3 weeks + 5 days from the first clinic visit). Following the completion of the second visit, two select sites also recruited participants who successfully completed the three-week study to participate in a cognitive interview to assess their comprehension of the PPIUS. This cognitive interview was designated as the third visit.

### Study questionnaires

#### Patient perception of intensity of urgency questionnaire (PPIUS)

**Figure 1:**
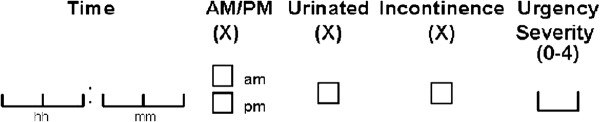
Urination episode entry field in the micturition diary.

The PPIUS is part of a daily micturition diary to be completed for three days in a given week. Participants were instructed to record for each urinary episode throughout the day: the time of urination, whether the episode was an urination (defined as passed urine into the toilet) or incontinence (defined as involuntary release of urine), and the “degree of associated urgency according to the definitions provided” (Figure 1). The PPIUS uses a 5-point scale for measurement of both urinary urgency and urge incontinence. Responses range from “No urgency” to “Mild urgency,” “Moderate urgency,” “Severe urgency,” and “Urge incontinence.” The definitions provided for the urgency ratings are listed in Table 1.

**Table 1:** Definition and scale of Patient Perception of Intensity of Urgency Scale (PPIUS)


**Urinated:**		Passed urine in the toilet.
**Incontinence:**		Involuntary release of urine.
**Urge Incontinence:**		Involuntary release of urine accompanied or immediately preceded by urgency
**Urgency:**		
0	No urgency	I felt no need to empty my bladder, but did so for other reasons.
1	Mild urgency	I could postpone voiding as long as necessary, without fear of wetting myself.
2	Moderate urgency	I could postpone voiding for a short while, without fear of wetting myself.
3	Severe urgency	I could not postpone voiding, but had to rush to the toilet in order not to wet myself.
4	Urge incontinence	I leaked before arriving to the toilet.

Patient- and clinician-completed overall treatment effect.

#### Patient- and clinician-completed overall treatment effect

The Overall Treatment Effect (OTE-Patient and OTE-Clinician) assesses patients’ change in symptoms since the previous study visit. The questions ask the patient/clinician to indicate whether OAB symptoms have improved, remained the same, or worsened since the last study visit.[10-12] The OTE-Patient and OTE-Clinician provided the information for classification of stable patients at the end of the three-week study in order to assess test-retest reliability.

#### RAND 36-item short form health questionnaire

The validated RAND 36-item short form questionnaire is a commonly used generic instrument designed to measure health-related quality of life [13]. The questionnaire consists of 36 items pertaining to the following eight health categories: physical functioning, social functioning, role limitations due to physical health problems, role limitations due to emotional problems, pain, mental health, vitality, and general health perceptions. The RAND-36 was administered at the first visit to characterize the initial characteristics of the study participants.

### Quantitative analysis

#### Descriptive statistics

Descriptive statistics were used to summarize demographic information collected from the participant and clinical information collected by the clinician at the first visit, as well as the number of micturition per 24 hours, number of incontinence episodes per 24 hours, and the PPIUS mean scores. Categorical variables derived from the OTE-Patient and the OTE-Clinician were also summarized. A micturition was defined as an entry in the diary with a checked “urinated” box. An incontinence episode was defined as an entry in the diary with checked “incontinence” box. If no “incontinence” box was checked for a given day, the number of incontinence episodes was set as zero for that day.

The daily PPIUS score was the mean of all reported urgency ratings on that day (including daytime and nighttime—a diary day starts when the subject gets up with the intention of staying awake and ends on the following day when the subject gets up). The mean level of urgency for a given week was the mean of the non-missing PPIUS daily scores over the three-day micturition diary period of the corresponding week.

#### Test-retest reliability

Data from participants whose OAB health state was classified as stable between the first and second clinic visits were used for assessment of reproducibility. Two definitions of stable patients were used and two separate analyses of reproducibility were performed. First, a stable patient was defined as those who responded “about the same,” “almost the same, hardly better at all,” or “almost the same, hardly worse at all” on the OTE-Patient [10-12]. Second, a stable patient was defined those who were classified as “about the same,” “almost the same, hardly better at all,” or “almost the same, hardly worse at all” based on the OTE-Clinician.

The assessment of reproducibility was conducted by comparing the stable patients’ PPIUS mean level of urgency during Week 2 and Week 3. The test-retest reliability was assessed through paired t-tests, Spearman correlation, and ICC, and it was assumed that the ICC should be greater than 0.80 which represented good to excellent test-retest reliability [14].

#### Acclimation effect

Acclimation effect of the PPIUS was assessed by comparing the mean level of urgency at Weeks 1, 2, and 3 of the stable patients defined based on the OTE-Patient. Paired t-tests were used to assess whether the PPIUS mean level of urgency was significantly different between Week 1 and Week 2, between Week 2 and Week 3, and between Week 1 and Week 3.

### Qualitative analysis

Cognitive interviews were conducted to gather feedback from the participants as an assessment of the content validity of the PPIUS. Participants were interviewed by a trained interviewer from United BioSource Corporation (UBC) using a standardized semi-structured interview guide with a think-aloud approach. The interview guide provided instructions for conducting the interview and for questioning the patients on their understanding of the PPIUS content and how they selected the PPIUS ratings. The interview guide included questions on the daily diary instructions, item content, and response scales. All interviews were audio recorded and transcribed.

Content analysis and descriptive statistics were used to analyze the responses collected during the cognitive interviews. Qualitative data was analyzed using ATLAS.ti software [15]. Using ATLAS.ti, qualitative data (patient quotes from interviews) were systematically analyzed, coded, and compared. “Open coding” was performed to fracture the data (i.e., passages from transcripts and notes) into smaller units and identifying concepts, themes, or recurring regularities that appeared within each interview. Axial coding was then used to connect categories and subcategories of the data. Specifically, each of the detailed codes or categories in open coding was connected based on their emergent theoretical linkages so that they represented specific instances of more general, abstract phenomena or processes.

## Results

### Patient disposition

A total of 81 respondents were screened for eligibility, and a total of 44 participants were eligible and consented for the study. Of the 44 consented participants, 43 participants completed the first clinic visit and 41 participants completed the second clinic visit. Forty-one participants completed three-day diaries for Week 1 and Week 2, however only 39 participants completed both clinical visits and all three weeks of the three-day diaries. These 39 participants were defined as the completers. Reasons for discontinuation after consent included screen failure due to positive urinary tract infection (UTI) dipstick test (n = 1), change in medication (n = 1), non-compliance with diary completion (n = 1), and loss to follow up (n = 2).

Twelve of the 39 successful completers of the test-retest study also completed the cognitive interview (third visit).

### Sociodemographic and clinical characteristics

Demographic and clinical characteristics reported by patients are summarized in Table 2 for the 39 participants who completed the test-retest study and the 12 successful completers who also completed the cognitive interviews. The mean (standard deviation [SD]) age of the 39 participants was 59.1 (15.23) years and most participants were Caucasian (n = 27, 69.2%), female (n = 32, 82.1%), and the most common patient-reported comorbid conditions were hypertension (n = 20, 51.3%) and arthritis (n = 17, 43.6%). Norm-based scores of RAND-36 were computed to a 0–100 score scale, where the average health of the general population is around 50 with a SD of 10 [16]. In general, the participants reported below-average physical health on the RAND-36, but average mental health (Table 2). 

**Table 2:** Participant-reported demographic characteristics—cognitive interview participants

	**Test-retest study completer**	**Cognitive interview completer**
**Characteristic**	**N = 39**	N = 12*
*Age (yrs)*		
Mean (SD)	59.1 (15.23)	61.6 (13.5)
*Gender, n (%)*		
Male	7 (17.9%)	1 (8.3%)
Female	32 (82.1%)	11 (91.7%)
*Race, n (%)*		
White	27 (69.2%)	8 (66.7%)
Black or African-American	3 (7.7%)	1 (8.3%)
Hispanic	3 (7.7%)	3 (25.0%)
Asian	6 (15.4%)	0 (0.0%)
*Employment status, n (%)*		
Full-Time	8 (20.5%)	2 (16.7%)
Part-Time	6 (15.4%)	1 (8.3%)
Retired	13 (33.3%)	7 (58.3%)
Disabled	5 (12.8%)	1 (8.3%)
Others	5 (12.8%)	0 (0.0%)
Missing	2 (5.1%)	1 (8.3%)
*Highest level of education, n (%)*		
Secondary/high school	10 (25.6%)	1 (8.3%)
Some college	17 (43.6%)	7 (58.3%)
Postgraduate degree	8 (20.5%)	2 (16.7%)
Other	4 (10.3%)	2 (16.7%)
*Comorbid conditions n (%)*		
None	9 (23.1%)	3 (25.0%)
Arthritis	17 (43.6%)	6 (50.0%)
Cancer	1 (2.6%)	1 (8.3%)
Diabetes	7 (17.9%)	2 (16.7%)
Hypertension	20 (51.3%)	5 (41.7%)
Others	7 (17.9%)	0 (0.0%)
*Length of time with bladder symptoms (yrs)*		
Mean (SD)	6.7 (6.78)	5.8 (4.6)
*RAND SF-36 (mean, SD)*		
Physical component summary (PCS)	43.9(11.62)	44.5(12.77)
Mental component summary (MCS)	49.4 (9.45)	51.9 (9.54)
Physical function	42.9(13.39)	42.4(13.68)
Role physical	44.8(12.10)	45.0(12.23)
Bodily pain	47.4(11.32)	47.4(11.07)
General health	45.8(10.13)	50.0 (7.09)
Vitality	48.6 (8.57)	53.4 (8.06)
Social functioning	46.8(10.63)	45.4(12.21)
Role emotional	45.3(11.58)	52.4 (8.14)
Mental health	49.7 (8.13)	50.0 (7.09)

*12 of the 39 test-retest study completers were also participants in a cognitive interview.

The mean (SD) overall age was 61.6 (13.5) years for the 12 cognitive interview participants. Most participants were Caucasian (n = 8, 66.7%), female (n = 11, 91.7%), and retired (n = 7, 58.3%), and the most common comorbid conditions reported by participants were arthritis (n = 6, 50%) and hypertension (n = 5, 41.7%). Participants reported experiencing bladder symptoms for an average of 5.8 years.

### Clinician-reported clinical characteristics

As assessed by clinic staff, the 39 participants had been patients at their respective clinics for a mean (SD) of 2.9 (2.4) years. Almost all participants’ (n = 35, 89.7%) primary urologic diagnosis was OAB; other primary diagnoses included urge incontinence (n = 2), benign prostatic hyperplasia (n = 1), and frequency (n = 1). Clinicians indicated most participants’ primary urinary complaint as frequency (n = 27, 78.1%), followed by urgency (n = 7, 17.9%). Based on the clinical chart, participants were being prescribed the following medications: oxybutynin (n = 12, 30.8%), solifenacin (n = 10, 25.6%), tolterodine (n = 6, 15.4%), darifenacin (n = 6, 15.4%), trospium (n = 4, 10.2%), and estradiol cream (n = 1, 2.6%).

Of the 12 participants that completed the cognitive interview, their primary clinician-reported urinary symptoms were frequency (n = 8, 66.7%), urgency (n = 3, 25.0%), and nocturia (n = 1, 8.3%). Secondary urinary complaints included urgency (n = 7, 58.3%), incontinence (n = 5, 41.7%), nocturia (n = 4, 33.3%), and frequency (n = 2, 16.7%) (participants could indicate more than one). The 12 participants were currently prescribed solifenacin (n = 4, 33.3%), oxybutynin (n = 4, 33.3%), tolterodine (n = 3, 25.0%), and darifenacin (n = 1, 8.3%).

### Quantitative results from the test-retest study (n = 39)

#### Urgency rating, micturition, and incontinence

The overall mean (SD) level of urgency was 2.0 (0.78) at Week 1, 1.9 (0.91) at Week 2, and 2.0 (0.95) at Week 3. The average ratings of urgency severity were stable over the three weeks.

The mean (SD) number of micturition per 24-hours was 10.0 (5.26) at Week 1, 10.3 (5.40) at Week 2, and 10.6 (5.70) at Week 3. The mean number of micturition per 24-hours was stable over the three weeks.

The mean number of incontinence episodes per 24-hour period was 2.2 (3.99) at Week 1, 2.0 (3.46) at Week 2, and 1.9 (4.16) at Week 3. Again, the mean number of incontinence episodes per 24-hours was stable over the three weeks.

#### Test-retest reliability

Thirty-four participants (87.2%) were classified as stable based on the OTE-Patient and 35 participants (89.7%) were classified as stable based on the OTE-Clinician. Test-retest reliability of the PPIUS three-day mean score in stable patients as classified based on the OTE-Patient from Week 2 to Week 3 is shown in Table 3, with an ICC of 0.95 and Spearman’s correlation of 0.89 (p < 0.0001). The difference in the mean values between Weeks 2 and 3 was not statistically significant (p = 0.3043). Similarly, test-retest reliability of the PPIUS mean level of urgency in stable patients defined by the OTE-Clinician from Week 2 to Week 3 was also high (Table 3), with an ICC of 0.95, and Spearman’s correlation of 0.89 (p < 0.0001). The difference in the mean values between Weeks 2 and 3 was not statistically significant (p = 0.2677).

**Table 3:** **Test-retest reliability: PPIUS 3-day average scores in stable patients **^**1 **^**week 2 to week 3 (n = 34) **^**2 **^

**PPIUS score**	**Mean (SD) Week 2**	**Mean (SD) Week 3**	**Difference**^**3**^**(SD)**	**T-value**	**P-value**	**Spearman’s r**^**4**^	**ICC**^**5**^
	**OTE-Patient**						
3-day Average Score	2.0 (0.91)	2.1 (0.94)	0.05 (0.294)	1.04	0.3043	0.89***	0.95
	**OTE-Clinician**						
3-day Average Score	2.0 (0.90)	2.1 (0.93)	0.06 (0.290)	1.13	0.2677	0.89***	0.95

^1^ Evaluated in stable patients defined as scores of -1, 0, or 1 by the OTE-Patient.

^2^ Patients who have average scores at both weeks.

^3^ Week 3 average score – Week 2 average.

^4^ Spearman rank order correlations; * p < 0.05; **p < 0.001; ***p < 0.0001.

^5^ Intra-class correlation coefficient.

#### Acclimation effect

No statistically significant differences were found in the three-day mean PPIUS scores of stable patients between Week 1 and Week 2 (difference = -0.05, t = -0.66, p = 0.5173), Week 2 and Week 3 (difference = 0.01, t = 0.08, p = 0.9348), or Week 1 and Week 3 (difference = 0.05, t = 1.04, p = 0.3043). These results suggested that the PPIUS did not demonstrate an acclimation effect in this study.

### Qualitative results from the cognitive interview (n = 12)

During the cognitive interviews, participants described their thought process for completing the three-day diary including the PPIUS. Participants were asked for their interpretation of the diary instructions and to describe their experience completing the three-day diary. Participants were asked about their understanding of the definitions of the urgency ratings and whether they were consistent with their own experiences and interpretation of the rating terms. Finally, the participants were asked about the formatting and feasibility of completing the diary on a daily basis for an extended period of time.

#### Diary instructions

Participants were asked to summarize the diary instructions for diary completion in their own words. Nine participants (75.0%) were able to accurately summarize the instructions. Half of participants (n = 6, 50.0%) felt that the instructions were clear and did not need any changes. Suggestions for modifying the instructions included clarifying the 12-hour clock as opposed to military time (n = 2, 16.7%) and providing more instructions about when to record episodes as “daytime” or “nighttime” (n = 3, 25.0%).

#### Overall experience

Participants were also asked to speak about their overall experience with the three-day diary. Ten participants (83.0%) said they had no difficulty completing the diary. Six participants (50.0%) expressed a positive opinion of the diary. Others (n = 2, 16.7%) reported that they had trouble remembering to complete the diary, found the diary inconvenient (n = 1, 8.3%), or were bothered by having to record urinations at night (n = 1, 8.3%).

#### Definition of urgency ratings

Participants were asked if the definitions of the urgency ratings were clear. Nine participants (75.0%) reported that the definition provided for “No urgency” was consistent with their interpretation of the phrase. Seven participants (58.3%) indicated that the definition of “Mild urgency” was consistent with the way they think of the phrase. Eight participants (66.7%) and nine participants (75.0%), respectively, found the definitions of “Moderate urgency” and “Severe urgency” to be consistent with their interpretation of the phrases. When asked about the definition provided for “Urge incontinence,” nine participants (75.0%) felt that the definition was consistent with their interpretation of the phrase. Notable suggestions came from two participants for simplifying the definition by changing the word “postpone” to “*delay*;” and revising the definition of “Moderate urgency” from “*I could postpone voiding ….without fear of wetting myself*” to “*I could postpone voiding for a longer period*.”

#### Formatting and feasibility

Because the participants rated their PPIUS rating on a micturition diary, they were also asked about the diary itself. Seven participants (58.3%) responded positively to the diary formatting and described it as “*wonderful*,” “*organized*,” “*good,*” “*self explanatory*,” “*great*,” and “*well done*.” Participants were then asked to describe a typical day when they completed the diary. Seven participants (58.3%) said they kept the diary by their bed at night. Six participants (50.0%) said they completed their diary entry after each episode, while three participants recorded the episode on a separate sheet of paper after each episode and transferred the information to the diary at a later time.

## Discussion

In order to assess content validity and test-retest reliability of the PPIUS, this study recruited OAB patients to complete a three-day micturition diary including the PPIUS for three consecutive weeks followed by cognitive interviews with participants at selected sites.

During the cognitive interview, participants were debriefed on the entire diary including the instructions, the urgency rating scale of the PPIUS, and their overall experience with the diary and completing it for three weeks. The interviews ascertained that participants interpreted the PPIUS as intended, with most participants reporting that they understood the urgency rating definitions, did not have difficulty rating their urgency grade for each micturition, and found the definitions to be consistent with their interpretation of the rating terms. Participants also reported that they liked and referred to the full urgency rating definitions that were included in the diary instruction packet.

Test-retest reliability of the PPIUS was assessed in 39 patients with OAB receiving pharmacological treatment for their symptoms. Reproducibility was excellent, as evidenced by high ICCs and Spearman’s correlations. In addition, paired t-tests found no difference of PPIUS three-day mean scores between any two weeks supporting that the PPIUS did not demonstrate an acclimation effect.

Although the PPIUS was based on the definition recommend by ICS Standardization of Terminology of Lower Urinary Tract Function Report [1] and the Committee for Proprietary Medical Products Standardization Sub-Committee [3], and has been used in other clinical studies [4-6], this is the first attempt in investigating the content validity of the PPIUS from the perspective of OAB patients. While cognitive interview participants were recruited from only two sites, the participants consisted of a good representation of age range, education level, comorbid conditions, and races. In addition, participants also were interviewed shortly after completing the diary for three consecutive weeks, to ensure participants could accurately recall their experience with the diary. The study results are representative and reliable.

Several limitations should be kept in mind when interpreting these results. First, previous studies have indicated that OAB was almost equally prevalent among men and women [17-20]. However, a relatively small percentage (18%) of men participated in the study, while target enrollment for men in OAB clinical trials is typically about 30%. The results could be more confidently generalized to the male patient population if more male patients were interviewed. Another limitation was that the urgency and incontinence continuum was not fully explored, which remained a challenge in assessing urinary urgency. One participant described experiencing incontinence at all level of urgency. Further studies are needed as Starkman et al [21]. pointed out “While some argue that urgency is episodic and maximal, we believe it can be subjectively graded. For example, some episodes of urgency can be suppressed, some persistently require immediate action, and some are so overwhelming that urge incontinence results. Although evidence for such a continuum is lacking, it remains conceptually intuitive.” PPIUS has been included in two completed, one withdrawn, and one ongoing clinical trial as shown in the ClinicalTrial.gov website.

## Conclusions

Participants in general demonstrated good understanding of the PPIUS instructions and found the ratings of urgency severity to be consistent with their interpretation of the ratings. Results of the cognitive interviews demonstrated evidence of content validity of the PPIUS, as participants in general agreed with the definition of the urgency ratings, finding the definitions consistent with their experiences. The PPIUS demonstrated excellent test-retest reliability as evidenced by high ICC in stable patients classified by both the OTE-Patient (ICC = 0.95) and the OTE-Clinician (ICC = 0.95). Acclimation effect was not observed among stable patients completing the PPIUS for three consecutive weeks. These findings support the use of the PPIUS as a reliable measure of urgency in both clinical trial and real life settings. The validity of PPIUS may be further established with future studies investigating the relationship between discretely graded urgency and incontinence continuum.

## Abbreviations

(ICC): Intra-Class Correlation Coefficient; (ICS): International Continence Society; (OAB): Overactive Bladder Syndrome; (OTE): Overall Treatment Effect; (PPIUS): Patient Perception of Intensity and Urgency Scale; (SD): Standard Deviation; (UBC): United BioSource Corporation; (UDS): Urodynamic Testing; (US): United States; (UTI): Urinary Tract Infection.

## Competing interests

WHC and DAR are employees of United BioSource Corporation and have research support from Astellas Pharma US and Astellas Pharma Global Development. SMN is a graduate student at NYU and was employed at United BioSource Corporation at the time the manuscript was completed. ML is an employee with Astellas Pharma Global Development, Deerfield, IL. ZH is an employee with Astellas Pharma Global Development, Leiderdorp, The Netherlands. IO is an employee with Astellas Pharma Europe Ltd, Staines, Middlesex, UK. TSM is an employee with Eli Lilly, and was employed with Astellas Pharma US, Deerfield, IL, at the time the manuscript was completed.

## Authors’ contributions

SMN and WHC participated in the collection and assembly of data, data analysis and interpretation, and manuscript writing. ML participated in data analysis and interpretation and manuscript writing. TSM, ZH, and DAR participated in the conception and design, interpretation, and manuscript writing. IO participated in the conception and design and manuscript writing. All authors read and approved the final manuscript.

## Pre-publication history

The pre-publication history for this paper can be accessed here:

http://www.biomedcentral.com/1471-2490/12/26/prepub

## References

[B1] Abrams P Artibani W Cardozo L Dmochowski R van Kerrebroeck P Sand P Reviewing the ICS 2002 terminology report: the ongoing debate Neurourol Urodyn 2009 28 287 10.1002/nau.20737 19350662

[B2] Coyne KS Payne C Bhattacharyya SK Revicki DA Thompson C Corey R The impact of urinary urgency and frequency on health-related quality of life in overactive bladder: results from a national community survey Value Health 2004 7 455 463 10.1111/j.1524-4733.2004.74008.x 15449637

[B3] EMA, EMA: Committee for Proprietary Medicinal Products Note for Guidance on the Clinical Investigation of Medicinal Products for the Treatment of Urinary Incontinence. CPMP/EWP/18/01 2002 London: EMA (European Medicines Agency)

[B4] Cardozo L Hessdorfer E Milani R Arano P Dewilde L Slack M Drogendijk T Wright M Bolodeoku J Solifenacin in the treatment of urgency and other symptoms of overactive bladder: results from a randomized, double-blind, placebo-controlled, rising-dose trial BJU Int 2008 102 1120 1127 10.1111/j.1464-410X.2008.07939.x 18990175

[B5] Dmochowski RR Newman DK Impact of overactive bladder on women in the United States: results of a national survey Curr Med Res Opin 2007 23 65 76 10.1185/030079907X159533 17257467

[B6] Chapple CR Martinez-Garcia R Selvaggi L Toozs-Hobson P Warnack W Drogendijk T Wright DM Bolodeoku J for the Ssg: A comparison of the efficacy and tolerability of solifenacin succinate and extended release tolterodine at treating overactive bladder syndrome: results of the STAR trial Eur Urol 2005 48 464 470 10.1016/j.eururo.2005.05.015 15990220

[B7] Cartwright R Srikrishna S Cardozo L Robinson D Validity and reliability of the patient's perception of intensity of urgency scale in overactive bladder BJU Int 2011 107 1612 1617 10.1111/j.1464-410X.2010.09684.x 21070569

[B8] Cartwright R Panayi D Cardozo L Khullar V Reliability and normal ranges for the Patient's Perception of Intensity of Urgency Scale in asymptomatic women BJU Int 2010 105 832 836 10.1111/j.1464-410X.2009.08846.x 19818081

[B9] Hays RD Revicki DA Assessing reliability and validity of measurement in clinical trials Quality of Life Assessment in Clinical Trials: Methods and Practice 2005

[B10] Jaeschke R Singer J Guyatt GH Measurement of health status. Ascertaining the minimal clinically important difference Control Clin Trials 1989 10 407 415 10.1016/0197-2456(89)90005-6 2691207

[B11] Matza LS Thompson CL Krasnow J Brewster-Jordan J Zyczynski T Coyne KS Test-retest reliability of four questionnaires for patients with overactive bladder: the overactive bladder questionnaire (OAB-q), patient perception of bladder condition (PPBC), urgency questionnaire (UQ), and the primary OAB symptom questionnaire (POSQ) Neurourol Urodyn 2005 24 215 225 10.1002/nau.20110 15747340

[B12] Juniper EF Guyatt GH Willan A Griffith LE Determining a minimal important change in disease-specific Quality of Life Questionnaire J Clin Epidemiol 1994 47 81 87 10.1016/0895-4356(94)90036-1 8283197

[B13] Ware JE Jr Sherbourne CD The MOS 36-item short-form health survey (SF-36). I. Conceptual framework and item selection Med Care 1992 30 473 483 10.1097/00005650-199206000-00002 1593914

[B14] Fleiss JL Statistical Methods for Rates and Proportions 1981 New York: Wiley

[B15] Muhr T User's Manual for ATLAS.ti 5.0, ATLAS 2004 Berlin: ti Scientific Software Development GmbH

[B16] Ware JE Jr Kosinski M SF-36 Physical & Mental Health Summary Scales: A manual for Users of Version 1 2001 2 Lincoln, RI: Inc. Q ed

[B17] Milsom I Stewart W Thuroff J The prevalence of overactive bladder Am J Manag Care 2000 6 S565 573 11183899

[B18] Milsom I Abrams P Cardozo L Roberts RG Thuroff J Wein AJ How widespread are the symptoms of an overactive bladder and how are they managed? A population-based prevalence study BJU Int 2001 87 760 766 11412210 10.1046/j.1464-410x.2001.02228.x

[B19] Stewart WF Van Rooyen JB Cundiff GW Abrams P Herzong AR Corey R Prevalence and burden of overactive bladder in the United States World J Urol 2003 20 327 336 12811491 10.1007/s00345-002-0301-4

[B20] Temml C Heidler S Ponholzer A Madersbacher S Prevalence of the overactive bladder syndrome by applying the International Continence Society definition Eur Urol 2005 48 622 627 10.1016/j.eururo.2005.04.026 15964133

[B21] Starkman JS Dmochowski RR Urgency assessment in the evaluation of overactive bladder (OAB) Neurourol Urodyn 2008 27 13 21 10.1002/nau.20472 17671973

